# *In Vivo* GFP Knockdown by Cationic Nanogel-siRNA Polyplexes

**DOI:** 10.3390/bioengineering2030160

**Published:** 2015-07-22

**Authors:** Arun R. Shrivats, Yuji Mishina, Saadyah Averick, Krzysztof Matyjaszewski, Jeffrey O. Hollinger

**Affiliations:** 1 Department of Biomedical Engineering, Carnegie Mellon University, 700 Technology Dr., Pittsburgh, PA 15219, USA; E-Mail: shrivats@alumni.cmu.edu; 2 School of Dentistry, University of Michigan, 1011 N. University Ave., Ann Arbor, MI 48109, USA; E-Mail: mishina@umich.edu; 3 Department of Chemistry, Carnegie Mellon University, 4400 Fifth Ave., Pittsburgh, PA 15213, USA; E-Mails: saverick@wpahs.org (S.A.); km3b@andrew.cmu.edu (K.M.)

**Keywords:** gene therapy, gene expression, RNA Interference, siRNA, GFP, ATRP, nanostructured polymer

## Abstract

RNA interference (RNAi) is a powerful tool to treat diseases and elucidate target gene function. Prior to clinical implementation, however, challenges including the safe, efficient and targeted delivery of siRNA must be addressed. Here, we report cationic nanogel nanostructured polymers (NSPs) prepared by atom transfer radical polymerization (ATRP) for *in vitro* and *in vivo* siRNA delivery in mammalian models. Outcomes from siRNA protection studies suggested that nanogel NSPs reduce enzymatic degradation of siRNA within polyplexes. Further, the methylation of siRNA may enhance nuclease resistance without compromising gene knockdown potency. NSP-mediated RNAi treatments against *Gapdh* significantly reduced GAPDH enzyme activity in mammalian cell culture models supplemented with 10% serum. Moreover, nanogel NSP-mediated siRNA delivery significantly inhibited *in vivo* GFP expression in a mouse model. GFP knockdown was siRNA sequence-dependent and facilitated by nanogel NSP carriers. Continued testing of NSP/siRNA compositions in disease models may produce important new therapeutic options for patient care.

## 1. Introduction

The induction of RNA interference (RNAi) is a powerful therapeutic tool to treat pathologies [1]. The clinical success of RNAi-based therapies, however, requires safe and efficient carriers for short interfering ribonucleic acids (siRNA). siRNA, when delivered to the cytoplasm of target cells, activate cellular machinery that bind and cleave messenger RNAs (mRNAs) with complementary sequences [2]. The clinical opportunities for RNAi are compelling; siRNA-based therapeutics may be designed to silence specific disease-causing genes enabling the treatment of diseases and genetic dysfunctions [3]. Current efforts with siRNA-based therapeutics span diseases including age-related macular degeneration [4], HIV [5] and various cancers [6,7,8]. RNAi, as a research tool, may also enable the discovery of new molecular signaling connections and the identification of biological roles and interactions for selected genes. The knockdown of target gene expression (both *in vitro* and *in vivo*) will impact cellular and tissue physiology and provide opportunities to elucidate pathogenesis and formulate treatments [9,10].

Due to the poor drug-like properties of siRNA (e.g., high molecular weight, electrostatic repulsion with cell membranes, degradation by nucleases, and rapid clearance from circulation), development as a therapeutic agent has been hampered [1,11,12,13,14]. The status quo for efficient siRNA delivery, namely, viral vectors, present both immunological and safety concerns, and are clinically unfavorable [15,16,17,18,19]. Polymer-based approaches for siRNA delivery are explored for their versatility [20]. Notably, the advent of controlled/living radical polymerizations has allowed for the production of highly uniform polymeric materials with complex structures, topology, composition and functionality [21,22].

Polymeric carriers for local siRNA delivery may employ a wide variety of cationic monomers to bind efficiently to anionic siRNA [23]. While this enables the transportation and protection of siRNA in physiological conditions, the strong cationic charge associated with these materials also produces a cytotoxic effect. Poly(ethylene oxide/glycol) (PEO/PEG) is frequently grafted onto cationic polymers to mask these charges and enable long-term circulation of polymer-siRNA complexes without detection by the immune system. This approach, however, has the well-known drawback of sacrificing the ability to internalize into desired cell types by reducing cationic charges, in order to reduce polymer toxicity (termed the “PEO/PEG dilemma”) [24].

With these challenges in mind, our groups have been investigating the development of biocompatible, stimuli-responsive polymeric carriers for local siRNA delivery. We have previously reported that cationic nanogels prepared by activators generated by electron transfer atom transfer radical polymerization (AGET ATRP) in inverse mini-emulsion can efficiently bind and deliver siRNA to knockdown Luciferase expression in a S2 drosophila cell-line [25]. The nanogels are approximately 300 nm in size with a Zeta potential of ~45 mV and are rendered biodegradable by virtue of a disulfide crosslinker enabling clearance these nanogels avoid the toxicity associated with cationic polymer carriers via the incorporation of a poly(ethylene oxide) shell. Nanogel NSP architecture is defined by a reducible cationic core surrounded PEO arms; nanogel NSPs distinguish themselves from traditional polymeric compositions by their ability to bypass physiological and cellular defenses and react to cytoplasmic stimuli to release an siRNA payload. A disulfide-based reducible core enables controlled polymer degradation and siRNA release in reductive microenvironments, such as the cell cytoplasm. The inclusion of hydrophilic cationic moieties—quaternized dimethylaminoethyl methacrylate (Q-DMAEMA)—enable siRNA binding while avoiding the polymer aggregation issues that have plagued traditional polymeric delivery systems [26]. Further, PEO arms enhance biocompatibility by partially masking surface cationic charges, and prevent enzymatic degradation of bound siRNA through steric hindrance [27].

We have reported that NSP complexed with siRNA show high biocompatibility with significant gene silencing of target genes in mouse osteoblasts and inhibiting osteoblast lineage progression [28,29]. *In vitro* biocompatibility of nanogel NSPs were confirmed up to NSP doses of 800 µg/mL in accordance with ASTM E2526 and ISO 10993-5 testing standards. *In vivo* NSP biocompatibility was determined up to 200 µg/scaffold by loading NSPs into XCM Biologic Tissue Matrix scaffolds (DePuy-Synthes) and implanting scaffolds in mouse hamstring muscles for four weeks [28]. Nanogel internalization, both with and without an siRNA payload, was confirmed an *in vitro* mammalian cell culture model [29]. These results also indicate that nanogel NSPs overcome the “PEO dilemma”, likely by the use of short PEO arms that ensure that nanogels maintain a net positive charge when complexed with siRNA [25].

In this reported study, we assessed cationic nanogels as a platform for gene knockdown in *in vitro* and *in vivo* mammalian models. Nanogel-siRNA polyplexes were assessed for stability in the presence of an endonuclease (RNaseA) and 2’-O-methylation of siRNA nucleotides was explored to improve resistance to nuclease degradation. Knockdown of GAPDH in a mammalian cell line was conducted in complete serum culture conditions. The ability of the cationic nanogel to deliver siRNA for gene knockdown *in vivo* was determined by GFP knockdown in a transient expression mouse model. From these studies, we report that cationic nanogel NSPs are an effective polymeric siRNA delivery system able to produce significant knockdown of target genes both *in vitro* and *in vivo*. The methylation of siRNAs also provides a means to enhance stability of siRNAs within nanogel polyplexes. Analyses of nanogel properties indicate a combination of high knockdown efficacy and biocompatibility, which are powerful antecedents for the production of a compelling genetic tool.

## 2. Experimental Section

### 2.1. Nanogel NSP Synthesis

We previously reported the synthesis and characterization of cationic nanogel NSPs and their potential for gene delivery [25]. In brief, cationic nanogels were prepared by activators generated by electron transfer atom transfer radical polymerization (AGET ATRP) in inverse miniemulsion by copolymerizing quaternized dimethyl aminoethylmethacrylate (qDMAEMA), oligo(ethylene oxide) methacrylate (OEOMA, M_n_ = 300), a water soluble disulfide methacrylate crosslinker (DMA) with a poly(ethylene glycol 2-bromoisobutyrate) (PEO_2k_iBBr) initiator and a copper bromide tris(2-(dimethylamino)ethyl)amine catalyst system dissolved in water. The inverse miniemulsion was prepared by ultra-sonication of the aqueous phase in a cyclohexane Span80 solution. After the reaction mixture was degassed an ascorbic acid solution was injected to generate the active catalyst. The cationic nanogels were purified by dialysis and characterized using dynamic light scattering and zeta potential analysis. Summary of the reaction ratios, ration conditions and nanogel final properties:PEO_2k_iBBr:OEOMA_300_:qDMAEMA:DMA:CuBr_2_:Ascorbic Acid: 1/290/20/4/0.5/0.6/0.3, 55 mg PEG_2k_, in 5% Span80 in cyclohexane for 24 hours at 30ºC. Size 275 nm PDI 0.164 Zeta potential 43.7 mV +/− 4.1. Further details on nanogel NSP synthesis and characterization can be found in the following reference [25].

### 2.2. Cell Culture

MC3T3 E1 Subclone 4 murine calvarial pre-osteoblasts (ATCC, Manassas, VA, CRL-2593) were cultured in alpha-minimum essential medium (α-MEM, Life Technologies, Carlsbad, CA, USA, A10490) supplemented with 10% fetal bovine serum (ATCC, 30-2020) and 1% penicillin/streptomycin (ATCC, 30-2300). When required, cells were passaged with 0.25% trypsin EDTA.

### 2.3. siRNA Protection Assay

siRNA complexation was determined by preparing weight:weight ratios of polymers with 500 ng siRNA in nuclease-free water (Ambion, Carlsbad, CA, AM9932). siRNA utilized in these studies included scrambled (unmethylated) sequences (Ambion, Carlsbad, CA, USA, AM4611) and selectively methylated siRNA against *Gapdh* and *Gfp*. Samples were incubated at 4 °C for 1 hour, after which glycerol (Sigma-Aldrich, St. Louis, MO, USA, G5516) was added to a final volume of 10% as a loading buffer. For siRNA protection studies, 0.1 U RNase A (Sigma-Aldrich, R4875) was added, and samples were incubated for 30 minutes at 37 °C prior to addition of glycerol. Polymer:siRNA solutions were loaded into 2% agarose (Sigma-Aldrich, A5304) gels in 1X TBE buffer (Bio-Rad Laboratories, Inc., Hercules, CA, USA, #161-0770EDU). A 100V potential was applied across gels for 30 minutes, after which they were stained in 0.5 μg/mL ethidium bromide (Sigma-Aldrich, E1510) in 1X TBE buffer for 1 hour. Gels were imaged under UV transillumination at 365 nm using an AlphaImager 2000 (Protein Simple, Santa Clara, CA, USA). siRNA band intensity was quantified using ImageJ (Bethesda, MD, USA).

### 2.4. In vitro GAPDH Knockdown

MC3T3 E1.4 cells were seeded at 25,000 cells/mL in a 96-well plate (0.2 mL per well). Polymer: siRNA treatments with nanogel NSPs were prepared and delivered to cells (n = 8) after 24 hours; *Gapdh* siRNA (Sense, selectively methylated: 5’-GGmUCmAUCmCAmUGAmCAACUUU-3’) was delivered at 20 pmol/50,000 cells. At all times, MC3T3 cells were cultured in complete α-MEM media containing 10% serum. Lipofectamine® RNAiMAX (Life Technologies, 13778030) treatments were prepared according to the manufacturer’s protocol. A media change was performed 2 days (48 hours) after initial seeding. The KDalert GAPDH Assay Kit (Life Technologies, AM639) was employed to evaluate changes in GADPH enzyme activity 3 days (72 hours) after siRNA treatments, as per the manufacturer’s protocol. Results were normalized to cells receiving no *Gapdh* siRNA treatment.

### 2.5. In vivo GFP Knockdown

10 μL of suspension containing 2.2 × 10^8^ PFU of recombinant Adenovirus that expresses GFP (Adex-GFP, Vector Development Laboratory, Baylor College of Medicine, Ad5-CMV-GFP) was injected to muscles in the lower hind limbs of wild type mice (mixed background of 129S6 and C57BL6). Hamilton syringe (model 1710SL, 100 uL SYR) and disposable needles (26sG, point style 2) (Hamilton Laboratory Products, Reno, NV, #81056 and #7758-02) were used and suspension was injected to left anterior tibial muscle from Achilles to the popliteal fossa. Sample sizes for each experiment are as indicated. Mice were euthanized three days later to observe fluorescence from dorsal and ventral sides of hind limbs. 2.2 × 10^8^ PFU per site of injection was the minimal amount of virus required for consistent levels of GFP fluorescence. 6 μL RNAi treatments were prepared using two different *Gfp* sequences (Sequence 1 Sense, with 3’ end nucleotides methylated: 5’-GCAAGCUGACCCUGAAGUUCAUmUmU-3’; Sequence 1 Sense, nucleotides selectively methylated: 5’-GmCAAGCmUGmACCCUmGAAGmUUmCAmU-3’; Sequence 2 Sense: 5’-GCACCAUCUUCUUCAAGGAdTdT-3’) and delivered at the time of Adex-GFP delivery. PBS treatments (no siRNA) were used as a control in contralateral hind limbs. The methodology behind the simultaneous delivery of Adex-GFP and RNAi treatments is supported by several published works [30,31,32]. Mean GFP fluorescence was measured using ImageJ grey value analysis function and compared between RNAi-treated limbs and contralateral limbs. All mouse experiments were performed in accordance with University of Michigan guidelines covering the humane care and use of animals in research.

### 2.6. Statistical Analyses 

For siRNA complexation analyses, unpaired Student’s *t*-tests were used to compare results between two populations (RNase-treated groups *vs.* control groups) and determine statistically significant differences between them (reported based on *p* < 0.05). GAPDH results were analyzed by ANOVA with Tukey’s *post-hoc* analysis; significant differences were reported based on *p* < 0.05. ANOVA was selected as a means of identifying statistically significant results among the seven experimental cohorts. GFP fluorescence was quantified by mean fluorescence (in both dorsal and ventral views) using ImageJ (Bethesda, MD, USA). Statistically significant results were determined by a paired Student’s *t*-test (*p* < 0.05) comparing mean fluorescence in treatment limbs *versus* contralateral control limbs. All statistical analyses were conducted with Minitab 16 (State College, PA, USA).

## 3. Results and Discussion

### 3.1. Results

**Figure 1:**
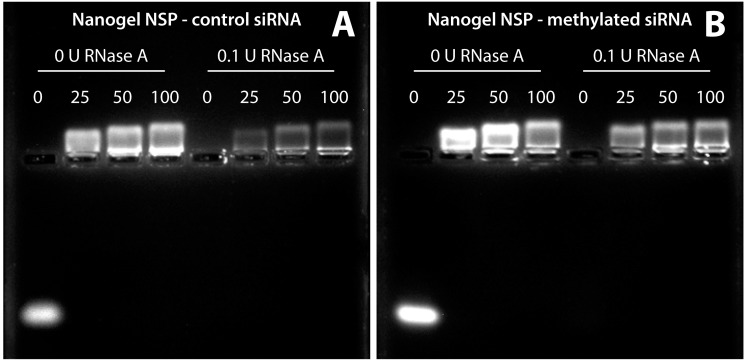
Evaluation of siRNA protection in the presence of a potent endonuclease, RNase A. Nanogel NSPs were complexed to control (**A**) or selectively methylated (**B**) siRNA and evaluated for polyplex stability by gel electrophoresis after incubation with 0 or 0.1 Units of RNase A. Polyplex complexation ratios (NSP:siRNA) were varied while siRNA quantities were held constant (500 ng/well). Nanogel NSPs impart protection on control siRNA, though the selective 2’O-methylation of siRNA appears to increase siRNA durability within polyplexes.

Nanogels at NSP:siRNA weight ratios of 25:1, 50:1 and 100:1 demonstrated efficacy in inhibiting degradation of siRNA (Figure 1A). 0:1 NSP:siRNA ratios (no nanogel carrier) cohorts were fully degraded (100%) after 30 minutes in the presence of RNase A. Nanogels prevented full siRNA degradation, producing 42.30 ± 4.81% decreases in siRNA staining intensity. Selectively 2’-O-methylated siRNA complexed with nanogel NSPs produced greater band intensity after incubation with RNase A as compared to control (unprotected) siRNA (Figure 1B). In contrast, staining intensity for selectively methylated siRNA-nanogel complexes decreased by 23.82 ± 9.99%. Data indicated significant increases in nuclease resistance of selectively methylated siRNA compared to unprotected siRNA (*p* < 0.05). 

**Figure 2:**
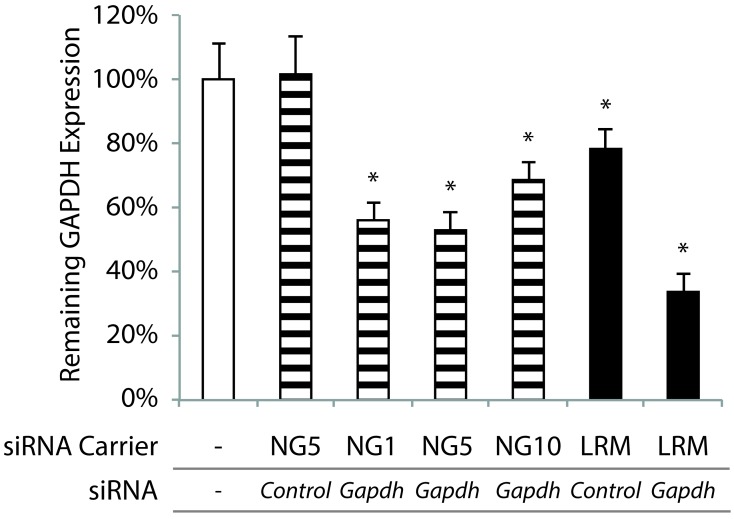
GAPDH knockdown in MC3T3 cells by siRNA delivery. Nanogel NSP groups resulted in significant knockdown of GAPDH expression as determined by the KDalert™ GAPDH assay. Nanogel 1:1 (NG1), 5:1 (NG5) and 10:1 (NG10) ratios achieved significant reductions in GAPDH expression, with 1:1 and 5:1 groups demonstrating peak knockdown. Gapdh siRNA delivered by Lipofectamine® RNAiMAX (LRM) achieved maximal knockdown of 70+%; however, control (scrambled) siRNA delivery by RNAiMAX also induced a reduction in GAPDH expression, indicating nonspecific gene silencing, potentially due to dose-dependent cytotoxicity of RNAiMAX. Nanogel NSPs (NG5) delivering control siRNA resulted in no significant differences in GAPDH expression. Data expressed as means (n = 8) + standard deviations. Asterisks indicate statistically significant differences from 100% *Gapdh* mRNA expression, determined by Tukey’s post-hoc analysis, based on *p* < 0.05.

GAPDH enzymatic activity was inhibited by nanogel-mediated *Gapdh* siRNA delivery to MC3T3 cells (Figure 2). Nanogel NSPs delivering siRNA at 1:1 (NG1) and 5:1 (NG5) ratios produced 43.9 ± 5.6% and 47.0 ± 5.5% GAPDH knockdown, respectively. Lipofectamine RNAiMAX induced a 66.3 ± 5.3% reduction in GAPDH when delivering *Gapdh* siRNA. However, a significant reduction in GAPDH activity of 21.7 ± 6.4% was detected when scrambled (nonspecific) siRNA sequences were delivered by Lipofectamine (*p* < 0.05). In contrast to Lipofectamine, the delivery of scrambled siRNA by nanogels produced negligible changes (1.5 ± 12.0%) in GAPDH activity, indicating minimal nonspecific gene silencing results.

**Figure 3:**
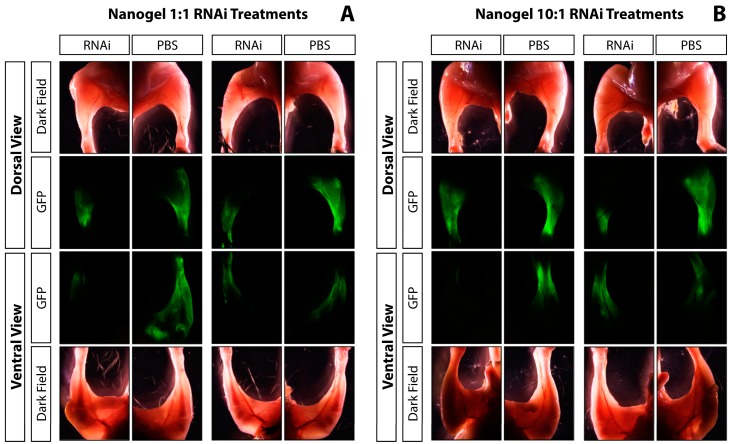
Ratiometric analysis of GFP knockdown by nanogel NSP. Nanogel NSPs were complexed with two μg of selectively methylated *Gfp* siRNA (sequence 1) at 1:1 (n = 8) or 10:1 (n = 7) ratios. Treatments were injected to left hind limbs along with 2.2 × 10^8^ PFU/site of Adex-GFP. Right hindlegs received the same amount of Adex-GFP mixed with the same volume of PBS. Levels of GFP were observed from dorsal and ventral sides. Photographs were taken from dorsal and ventral sides and levels of fluorescence were quantified using ImageJ grey value analysis function. Left panel: Mean GFP expression in NG1:1 RNAi-treated limbs was 2.80 ± 2.05% and in control (PBS-treated) limbs was 6.07 ± 1.68% (*p* = 0.002). Right panel: Mean GFP expression in NG10:1 RNAi-treated limbs was 2.98 ± 1.83% and in control limbs was 4.58 ± 2.27% (*p* = 0.209).

**Figure 4:**
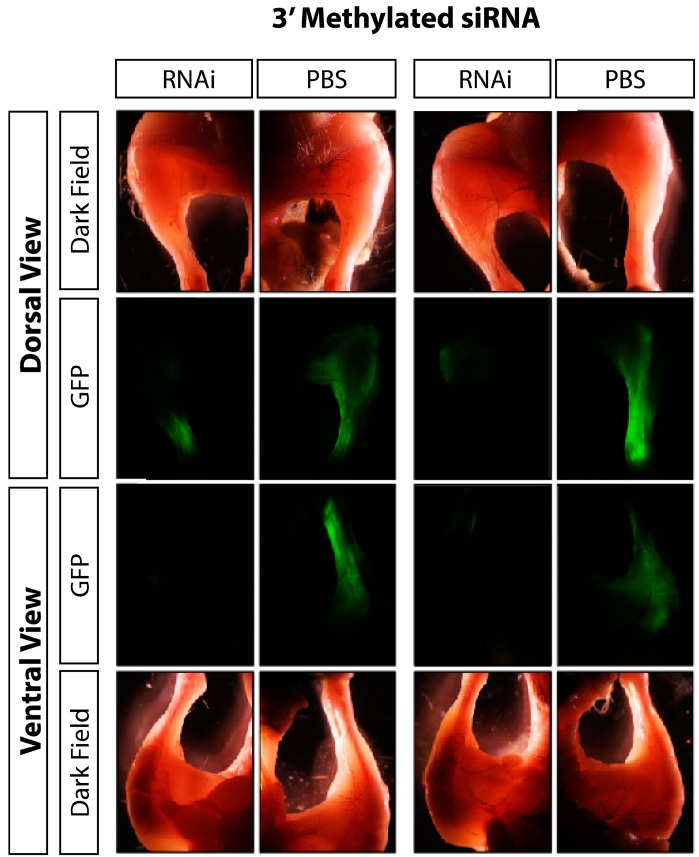
Efficacy of RNAi treatments with 3’ methylated *Gfp* siRNA. Nanogel NSPs were complexed at 1:1 ratios with two μg of 3’ methylated (n = 5) siRNA (sequence 1). Treatments were injected to left hind limbs along with 2.2 × 10^8^ PFU/site of Adex-GFP. Right hindlegs received the same amount of Adex-GFP mixed with the same volume of PBS. Levels of GFP were observed from dorsal and ventral sides. Mean GFP expression in RNAi-treated limbs was 2.75 ± 1.24% and mean expression in control (PBS-treated) limbs was 5.99 ± 1.41% (*p* = 0.011).

Nanogel NSPs produced consistent gene knockdown in a transient GFP mouse model. Nanogel NSPs delivering selectively methylated anti-*Gfp* siRNA (*Gfp* Seq. 1) at 1:1 ratios suppressed GFP expression relative to control limbs (Figure 3-left). Grey value quantitation of fluorescence in PBS-treated limbs was 6.07 ± 1.68 (arbitrary units). Contralateral limbs receiving 1:1 *Gfp* siRNA treatments exhibited fluorescence levels of 2.80 ± 1.05 (n = 8, *p* = 0.002), correlating to 53.9% knockdown. Mean fluorescence of nanogel 10:1 treated limbs was 2.98 ± 1.83 *versus* 4.58 ± 2.27 in PBS-treated limbs, correlating to 34.9% knockdown (n = 8, *p* = 0.21) (Figure 3-right). Delivery of 3’-methylated siRNA by nanogel NSP at 1:1 ratios efficiently suppressed GFP expression (Figure 4). Mean fluorescence in RNAi-treated limbs was 2.75 ± 1.24 *versus* 5.99 ± 1.41 in PBS-treated limbs (*p* = 0.011)—a reduction of 54.1%. There were no significant differences in GFP knockdown between selectively methylated siRNAs and 3’-methylated siRNAs. 

**Figure 5:**
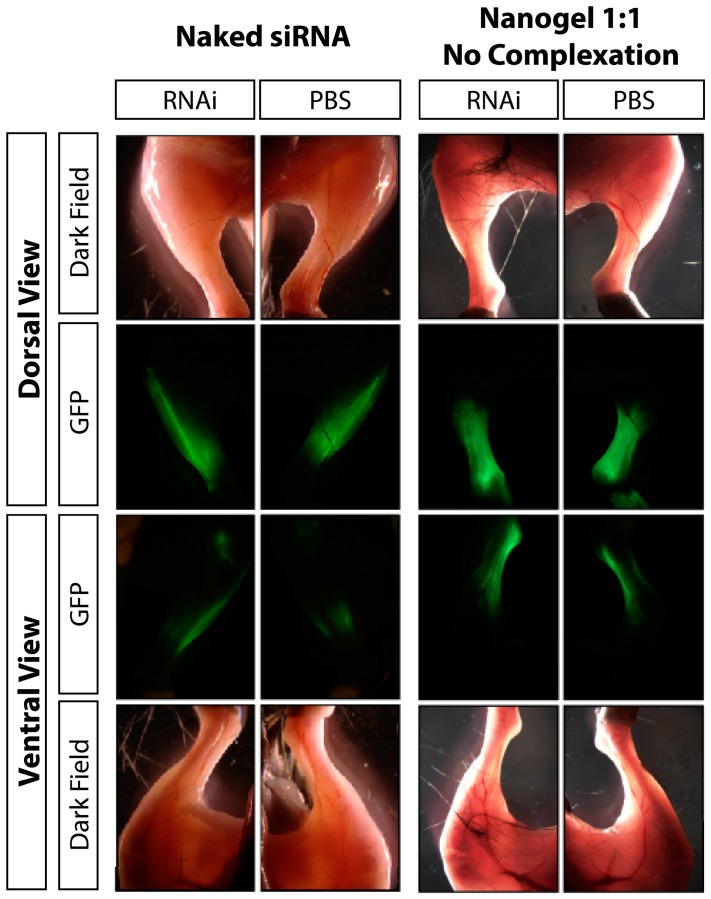
Evaluation of naked siRNA delivery and the requirement for nanogel complexation. Left panel: two μg of 3’-methylated *Gfp* siRNA (without nanogel NSPs) were injected to hind limbs along with 2.2 × 10^8^ PFU/site of Adex-GFP as indicated in each row. Contralateral limbs received Adex-GFP and were treated with PBS. Reductions in GFP expression were not observed (n = 6, *p* = 0.811). Right panel: RNAi treatment limbs were treated with neutral nanogel NSP (no siRNA complexation) (n = 7, *p* = 0.339). Results confirm the requirement of complexation of siRNA to facilitate delivery to cells.

**Figure 6:**
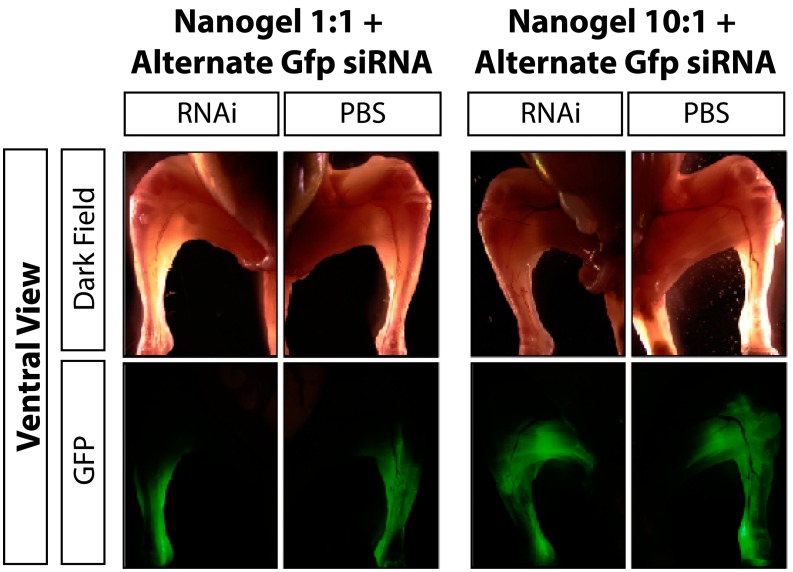
GFP knockdown by delivery of an alternate GFP siRNA sequence. Two μg of 3’-methylated siRNA for GFP (sequence 2) complexed with nanogel NSPs at 1:1 and 10:1 weight ratios (n = 6 and n = 7, respectively) were injected to left hind limbs along with 2.2 × 10^8^ PFU/site of Adex-GFP. Contralateral limbs received PBS treatments. There were no indications of reduced GFP expression (*p* = 0.567 and *p* = 0.428, respectively), indicating the lack of efficacy of this siRNA sequence. Results indicate that nanogel-mediated gene silencing is governed by the siRNA sequence, suggesting a targeted silencing effect dictated by siRNA sequence design.

The delivery of anti-*Gfp* siRNA without an NSP carrier (Figure 5-left) did not produce reductions in fluorescence compared to control limbs (n = 8, *p* = 0.811). Moreover, injection of selectively methylated *Gfp* siRNA with neutrally-charged NSPs without undergoing a complexation process (Figure 5-right) did not elicit significant reductions in GFP fluorescence either (n = 8, *p* = 0.339). An alternate anti-*Gfp* siRNA sequence (Seq. 2) was produced based on complementarity to the *Gfp* sequence. NSP delivery of anti-*Gfp* siRNA (sequence 2) did not produce knockdown (Figure 6). The nanogel 1:1 and 10:1 ratios were not effective against GFP expression (n = 8, *p* = 0.567 and *p* = 0.428, respectively). This is a strong indicator that nanogel-mediated gene silencing is sequence-dependent.

### 3.2. Discussion

Despite the notable barriers to successful siRNA delivery and gene knockdown, results indicate that nanogel NSPs combined with methylated siRNAs are able to navigate the *in vitro* and *in vivo* microenvironments and produce a potent, targeted gene knockdown effect. A key barrier with *in vitro* and *in vivo* gene knockdown paradigms is the degradation of siRNA by serum nucleases. Consequently, polymeric RNAi treatments are often delivered in serum-free *in vitro* culture conditions [13,33,34,35]. This is an inadequate analog to *in vivo* siRNA delivery. Imparting resistance of an RNAi therapeutic to nuclease degradation may be achieved by: (1) the siRNA carrier, and (2) the siRNA. Here, we combined both strategies by complexing a cationic, biocompatible nanogel synthesized by ATRP with selectively methylated siRNA, and produced a potent tool for *in vitro* and *in vivo* gene silencing.

The design and synthesis of nanogel NSPs requires attention to biocompatibility, siRNA complexation and protection, and targeted gene knockdown [25]. We sought to complement the protective capabilities of nanogel NSPs with improvements to the siRNA. An analysis of nanogel NSP protection of control (unmethylated) siRNA by gel shift assay suggested nanogel NSPs exhibited partial interference with RNase A, a potent endonuclease. It has been reported that chemical modification of siRNA base pairs may improve nuclease resistance and enable the production of RNAi treatments with increased potency *in vivo* [36]. However, chemical modification of siRNA may impede their gene silencing capacity. Thus, we exploited the 2’-O-methylation of siRNA to improve nuclease resistance when complexed to nanogel NSPs. Results suggested that methylation of siRNA diminished enzymatic degradation caused by incubation with RNase A. Through these assays, it was observed that NSP:siRNA ratios ranging from 25:1 to 100:1 were optimal to validate siRNA protection via gel assay, while ratios between 1:1 and 10:1 produced targeted gene knockdown effects in later assays. The necessity for increased NSP quantities during gel assays was primarily attributed to the superphysiological forces *(i.e.*, a potential difference of 100V) used to induce separation of polymers and siRNA in the gel assay. Consequently, nanogel NSPs and siRNAs that complex in physiological conditions may be separated by a potential difference of 100V, thus invalidating an assessment of nanogel protective capabilities. To compensate for this, nanogel content was increased during gel assays such that polyplexes remained intact in the gel environment, allowing for an assessment of siRNA degradation.

We previously reported nanogel-mediated gene knockdown of *Gapdh* mRNA in mammalian cell cultures [28]. However, gene expression may be regulated at a translational level and thus, analyses of protein expression/activity are indicative of successful gene silencing. Here, we extended analysis to the silencing of GAPDH expression (at a protein level) in MC3T3 cells. The selection of calvarial preosteoblasts was based on our validation of it as a robust platform for gene knockdown assays through our previous reported efforts to produce an RNAi prophylaxis for heterotopic ossification [29,37]. RNAi treatments against *Gapdh* were delivered by nanogel NSPs and compared to Lipofectamine RNAiMAX—a commercially available siRNA delivery reagent (Figure 2). Selectively methylated siRNAs significantly inhibited GAPDH activity when delivered by both nanogel and Lipofectamine carriers. The delivery of scrambled (nonspecific) siRNA via Lipofectamine, however, produced a significant inhibitory effect on GAPDH expression. Nonspecific GAPDH knockdown caused by Lipofectamine delivery suggests a potential reduction in cell viability. Knockdown metrics rely on intracellular controls; thus, decreases in cell viability may produce a perceived knockdown effect [38,39,40,41]. Nanogel NSPs, in contrast, did not produce any indications of nonspecific effects when delivered with control siRNA; when nanogels delivered *Gapdh* siRNA, treatments produced successful GAPDH knockdown in the presence of 10% serum. 

The progression from *in vitro* to *in vivo* presents challenges for siRNA delivery systems, largely due to added complexity in the kinetics and physiology of the local milieu. Consequently, it is necessary to re-calibrate NSP:siRNA delivery parameters *in vivo*. Thus, we used a GFP mouse model (using the Ad5-CMV-GFP) to determine gene silencing capabilities *in vivo*. 

The Ad5-CMV platform has been well-characterized for safety and transgene expression [42,43]. The simultaneous delivery of a GFP inducing agent and silencing agent is supported by multiple gene knockdown protocols [30,31,32]. Despite this support, there exists a need to further investigate the effects of varying the delivery times of the virus relative to siRNA. It is logical to suggest that delivery of the virus should not precede siRNA delivery significantly in order to minimize accumulation of target mRNA and proteins prior to siRNA delivery, that could mask any knockdown effects due to the potential long half-lives of the target proteins and the relative instability of siRNAs. This parameter would likely need to be optimized for each individual siRNA delivery platform; we recognize the need to further optimize this parameter. However, since that was not in the scope of our research, we based our protocol on the reported success of other studies based on simultaneous delivery of both the virus and siRNA [30,31,32]. Results suggested that nanogel RNAi treatments using both selectively methylated and 3’ methylated *Gfp* siRNA inhibited local expression of GFP *in vivo*. RNAi treatments featuring nanogels at 1:1 ratios produced consistent GFP knockdown in comparison to 10:1 ratios. Selective and 3’-methylation of anti-*Gfp* siRNA produced significant reductions in GFP expression, reflecting the therapeutic potential of the techniques. The delivery of naked siRNA produced negligible reductions in GFP expression, emphasizing that successful induction of RNAi in the GFP mouse model necessitated an siRNA delivery system. Non-complexing nanogel NSPs delivering anti-*Gfp* siRNAs did not produce a significant reduction in GFP expression. The delivery of an alternate *Gfp* siRNA sequence demonstrated the siRNA sequence-dependent nature of the GFP knockdown. These results further illustrate that silencing effects were dependent on the siRNA sequence delivered, and were not influenced by the presence of nanogel NSPs. The data reported underscore the potential for nanogel NSP-mediated siRNA delivery and gene silencing, both *in vitro* and *in vivo*. Nanogel NSPs facilitated sequence-dependent gene silencing in both transient and stably expressed gene knockdown models.

## 4. Conclusions

Nanogel NSPs were synthesized by ATRP for the delivery of siRNA for gene silencing. The outcome from this tactic is siRNA protection, and efficient siRNA delivery *in vitro* and *in vivo*. Steric hindrance by nanogel NSPs, and 2’-O-methylation of siRNAs resulted in the maintenance of polyplex integrity in the presence of RNase A. Nanogel NSPs facilitated knockdown of GAPDH in a full serum mammalian cell culture model, and of GFP in a GFP mouse model. Knockdown results included genetic targets that were constitutively active (*Gapdh*) and transiently upregulated (*Gfp*) both *in vitro* and *in vivo*. The gene silencing measured was sequence-specific and dependent on cationic nanogel NSPs for efficient siRNA delivery. The results emphasize the potential for bioresponsive, biocompatible polymeric carriers for siRNA delivery to produce a compelling therapeutic genetic tool.
